# Fueling Plankton Production by a Meandering Frontal Jet: A Case Study for the Alboran Sea (Western Mediterranean)

**DOI:** 10.1371/journal.pone.0111482

**Published:** 2014-11-05

**Authors:** Temel Oguz, Diego Macias, Jesus Garcia-Lafuente, Ananda Pascual, Joaquin Tintore

**Affiliations:** 1 SOCIB, Balearic Islands Coastal Ocean Observing and Forecasting System, Palma de Mallorca, Spain; 2 Institute of Marine Sciences, Middle East Technical University, Erdemli, Mersin, Turkey; 3 Institute for Environment and Sustainability, Joint Research Center, European Commission, Ispra, Italy; 4 Departamento de Física Aplicada II, Universidad de Málaga, Málaga, Spain; 5 IMEDEA (CSIC-UIB), Esporles, Palma de Mallorca, Spain; University of Vigo, Spain

## Abstract

A three dimensional biophysical model was employed to illustrate the biological impacts of a meandering frontal jet, in terms of efficiency and persistency of the autotrophic frontal production, in marginal and semi-enclosed seas. We used the Alboran Sea of the Western Mediterranean as a case study. Here, a frontal jet with a width of 15–20 km, characterized by the relatively low density Atlantic water mass, flows eastward within the upper 100 m as a marked meandering current around the western and the eastern anticyclonic gyres prior to its attachment to the North African shelf/slope topography of the Algerian basin. Its inherent nonlinearity leads to the development of a strong ageostrophic cross-frontal circulation that supplies nutrients into the nutrient-starved euphotic layer and stimulates phytoplankton growth along the jet. Biological production is larger in the western part of the basin and decreases eastwards with the gradual weakening of the jet. The higher production at the subsurface levels suggests that the Alboran Sea is likely more productive than predicted by the satellite chlorophyll data. The Mediterranean water mass away from the jet and the interiors of the western and eastern anticyclonic gyres remain unproductive.

## Introduction

**Figure 1:**
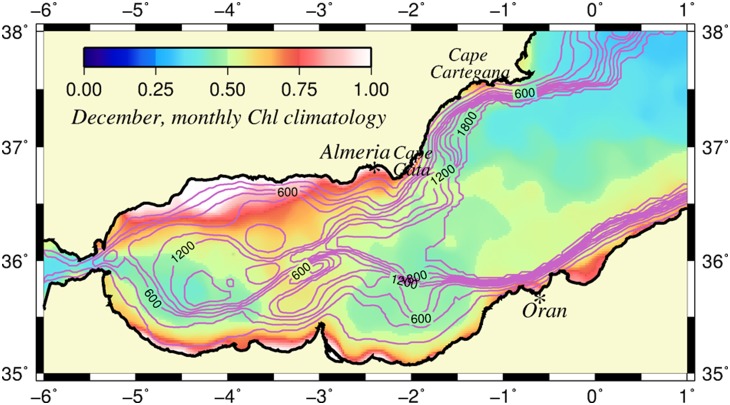
Satellite-derived chlorophyll distribution. The climatological December surface chlorophyll concentration distribution (mg m^−3^), as well as the smoothed model topography used in the model (contour interval is 200 m). The chlorophyll data were retrieved from the 9 km gridded monthly SeaWiFS products, and displays a relatively oligotrophic WAG and EAG and more productive zone along their peripheries, as well as the southern and northern coasts. They were separated from relatively low concentrations of the Algerian basin by a well-defined front. The December monthly climatology was chosen particularly because it did not include an additional impact of winter mixing on chlorophyll distribution.

Frontal zones are known to be sites of enhanced primary production, providing the fertilization of surface water, high planktonic biomass accumulation, and an efficient biological pump particularly for oligotrophic marine environments. The Alboran Sea (Fig. 1), located at the western end of the Mediterranean Sea and connected to the Gulf of Cadiz in the Eastern Atlantic Ocean through the Gibraltar Strait (hereinafter also referred to as “strait”), constitutes an ideal test basin for studying the biological impacts of frontal processes [1]. The predominant circulation pattern within the upper 150–200 m layer of the Alboran Sea involves an incoming meandering buoyant jet from the Gulf of Cadiz through the Gibraltar Strait and either both the Western Alboran Gyre (WAG) and the Eastern Alboran Gyre (EAG) with a cyclonic area in between or only the WAG [2]–[5]. The jet is characterized by a well-defined frontal zone with a density contrast up to 1.0 kg m^−3^ between more dense waters located to the left of the jet and less dense waters to the right [6]–[9]. Less frequent patterns of the circulation system involve the absence of gyres [2] or the simultaneous presence of three anticyclonic gyres [2], [10].

The average annual mean primary productivity of the Alboran Sea exceeds 100 gC m^−2^ yr^−1^ [11], comparable to those observed in mesotrophic systems and among the highest within the oligotrophic Mediterranean Sea. A major part of the plankton productivity (>300 gC m^−2^ yr^−1^) appears to be associated with the Atlantic jet, as documented by observations performed along the frontal zone around the WAG [12]–[14], the EAG [6], [15]–[17] and along the Algerian coast [18], [19]. The link between the frontal jet structure and phytoplankton production is so robust and persistent that it may appear in the form of a narrow zone of relatively high chlorophyll concentrations around the anticyclonic gyres even in the long-term mean winter ocean color data (Fig. 1).

The roles played by different dynamical processes on the biogeochemical characteristics of the Alboran Sea have not yet been quantified. Excluding the models that treated the Alboran Sea as a part of the western or the whole Mediterranean Sea [20], [21], the only coupled physical-biochemical modeling effort dedicated to the biochemical-physical interactions in the Alboran Sea [22] simulated the annual plankton production cycle in reasonable agreement with the observations system, but it did not explore the specific role of frontal dynamics. Following the objectives of the SOCIB (Balearic Islands Coastal Ocean Observing System) Strategic Plan [23], the present process study puts forth the meandering frontal jet to control the first order dynamics of the physical-biological interactions governing the observed biological production characteristics of the Alboran Sea. The individual roles of other physical factors (such as wind, strong winter turbulence mixing, and modulation of the Atlantic jet by tidal and subinertial variability within the Gibraltar Strait, etc.) were not addressed here and were left for a future study.

The following provides a brief description of the biophysical model and its implementation relative to the Alboran Sea conditions in section 2; the results of a particular model simulation in section 3; and a discussion on the main model findings, their comparison with the observations, and likely contributions of other mechanisms that are not included in the present study in section 4. The simulation described in the present study is not intended to represent the mean state of the circulation field. Instead, it demonstrates the capability of a jet-double gyres circulation system to promote enhanced biological production similar to the case shown in Fig. 1. The circulation system considered likely prevails at least for several months of the year, as depicted by the observations [4] and the model simulations [5]. Other forms of the circulation system may be associated with different forms of plankton production, as inferred by the satellite-derived surface chlorophyll concentration distributions. Various examples of these distributions are documented by [24] and http://www.socib.eu/index.php?seccion=modelling&facility=satimages&img=oceancolorwmed.

## Model Description

An eddy-resolving version of the Princeton Ocean Circulation Model (POM) was configured for the Alboran Sea and the western Algerian basin of the Western Mediterranean. The POM is a free surface, primitive equations model based on the f-plane, Boussinesq and hydrostatic approximations. The model domain extended from 6°W to 1.6°E and from 35°N to 39°N and was separated from the rest of the Mediterranean Sea by the open boundaries along its northern and eastern limits. Its western open boundary was located within the Gulf of Cadiz (Fig. 1). The model domain was discretized by an eddy resolving rectangular grid of 0.04° (3150 m) in the zonal direction and 0.025° (2775 m) in the meridional direction. It employed the terrain-following sigma coordinate in the vertical with 35 unevenly distributed levels, employing finer spacing near the surface and bottom [25].

The biogeochemical model, embedded on-line into the POM, comprised the single phytoplankton (P), zooplankton (Z), particulate organic (D), and dissolved inorganic nitrogen (N) groups, with nitrogen being the main limiting nutrient for this region [26]. This NPZD type biological model is particularly adequate for winter conditions because it is the most productive season [27], dominated mainly by the herbivorous food chain, with diatoms and copepods being the key functional groups growing under relatively low light and temperature and high nutrient conditions [15], [26], [28]. In addition, the choice of a more sophisticated multi-compartmental model (such as the one given by Oguz et al. [29]) does not provide any additional advantage for the problem studied here. A brief description of the initial and boundary conditions and the simplifications and idealizations introduced during the model formulation are provided below. A more detailed description of the biophysical model and the parameters’ setting are provided in the extended model description section below.

The wind stress and the total heat flux at the surface were set to zero to exclude the likely effects of wind- and buoyancy-induced vertical mixing, as well as the coastal upwelling/downwelling events on plankton production. The nitrate content of the initial nitrate profile within the upper 100 m layer was also set to zero in order to minimize the contribution of initially available nitrate for supporting plankton production. In addition, the advection of biological fields along the Gibraltar Strait were switched off to exclude the lateral supply of nutrients and biogenic material from the Gibraltar Strait. Under these conditions, plankton production within the Alboran Sea originated solely from the diapycnal advective and diffusive fluxes provided from deeper levels by the quasigeostrophic and ageostrophic processes. In the subsequent section, we demonstrated that the ageostrophic processes dominate within the frontal jet and lead to strong plankton production, whereas the quasigeostrophic processes predominated away from the immediate vicinity of the frontal jet.

The circulation field of the Alboran Sea developed from the initial state of rest and the horizontally uniform initial water mass representative of the winter mean conditions of the Medar/Medatlas climatology by prescribing the steady barotropic transport and the relatively warmer and less saline Atlantic water mass within the upper 200 m layer at the western open boundary. Similar inflow conditions were also specified across the northern boundary to incorporate the likely effects of the southward flowing Northern current crossing the Ibizia channel and reaching the Alboran Sea. The total flow (the baroclinic flow) across the open boundaries was treated by the free radiation condition in response to the evolving baroclinic structure of the flow field. Hence, in our model, the upper and lower layer transports within the strait were not directly imposed. Instead, they were obtained from the evolving thermohaline structure of the system and the prescribed barotropic transport at the western open boundary. For the present simulation, the upper and lower layer transports at the eastern exit section of the Gibraltar Strait to the Alboran Sea amounted to approximately 0.85 and −0.65 Sv, respectively. The upper layer transport was comparable with its climatological measured value, whereas the lower layer transport was slightly smaller but within the range of its observed variability [30], [31]. This setting had the advantage of showing that even relatively weak upper and lower layer transports across the Gibraltar Strait (representing a worst case scenario) can lead to enhanced frontal production within the Alboran Sea. Simulations with greater layer transport did not alter the main features of the emerging plankton production characteristics.

## Physical and Biological Characteristics of the Circulation System

### 1. Physical characteristics

Starting from an initially quiescent state and a horizontally uniform density field, the model was integrated for 120 days to obtain its dynamical adjustment to geostrophy (first 30 days), and then its temporal evolution (between days 30 and 90) to the fully nonlinear regime (after day 90). The physical and biological characteristics described below correspond to the latter nonlinear regime that, among different sensitivity experiments conducted within the framework of this study, resembled the jet-double gyres circulation system and the associated chlorophyll distribution similar to the one shown in Fig. 1.

**Figure 2:**
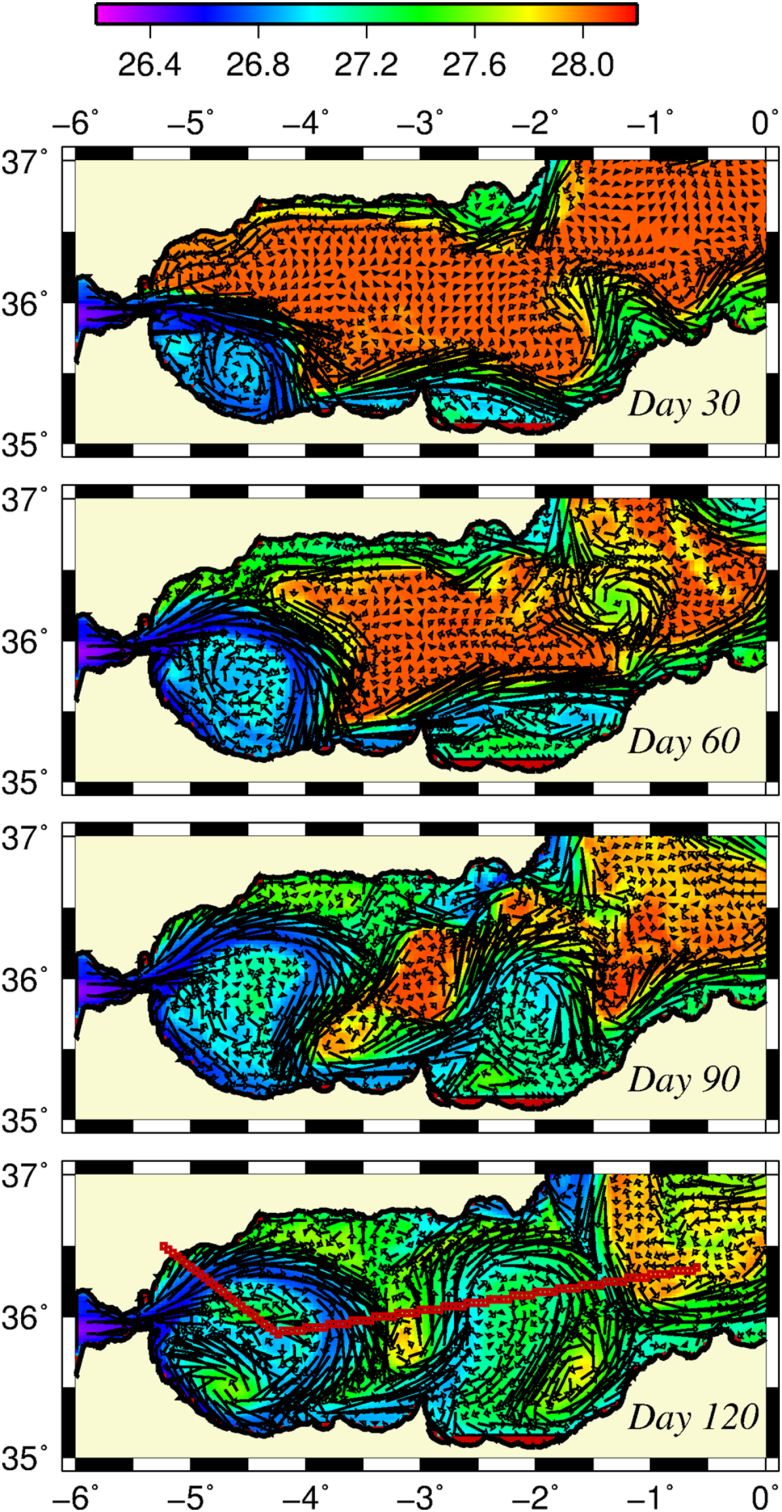
Surface density and currents in model simulation. Horizontal distribution of surface density (kg m^−3^) and flow field (m s^−1^) at days 30 (a), 60 (b), 90 (c), and 120 (d). The line shown in the last plot shows the position of the transect used in other figures. The arrows are scaled by 1 cm = 0.5 cm s^−1.^

In the early geostrophic adjustment phase, upon entering from the Gibraltar Strait with a current speed as high as 1.0 m s^−1^ and surface density ∼26.5 kg m^−3^, the positively buoyant fresher water jet was deflected to the south and continued to flow along the south coast under the action of the Coriolis force, advecting the lower salinity Atlantic water in the direction of Kelvin wave propagation (Fig. 2a). The jet exhibited three meanders: near the Gibraltar exit section; downstream of the Cape Tres Forcas; and along the SW-NE-oriented coastline. A similar dynamic of Kelvin wave propagation also took place along the Spanish coast in response to the coastal current system developed by the inflow across the northern open boundary. It flowed southward first and then westward with meander developments to the south of Cape Cartagena and near Cape Gata. The meanders and eddies along both coasts were triggered by the coastline irregularities and the topographic beta effect [34]–[36]. The increasing curvature of the southern coastline leads to a stronger offshore deflection and cross-isobath flow and a more pronounced flow instability. This early evolution was very similar to that presented in previous works [32], [33] to which we refer for further details.

During the subsequent month (Fig. 2b), the anticyclonic eddy along the SW-NE-oriented African coast was detached and gradually dissipated within the basin. The anticyclonic gyre at the Gibraltar exit section expanded gradually northward. The Atlantic jet entering the basin as a zonal jet fed this gyre and flowed around it to the south coast. It then proceeded mainly eastward, whereas a weak branch turned westward to close the gyre circulation. Later (Fig. 2c), the orientation of the Atlantic jet shifted gradually towards the northeast and the anticyclone near the Gibraltar exit section evolved to form a well-established WAG structure covering the basin up to 3.5°W longitude. Similarly, the coastal anticyclone to the east of Cape Tres Forcas expanded offshore (Fig. 2b) and evolved into a meridionally elongated EAG between 2.5°W and 1.0°W (Fig. 2c). The meanders of the northern coast were eventually detached and made the westward boundary current along the Spanish coast that is less well-depicted due to its interaction with the gyres. The region between the WAG and EAG was occupied by a cyclonic circulation (Fig. 2c).

The WAG grew further toward the northern coast and eastward during the rest of the integration period (up to day 120) (Fig. 2d). The steady growth was related to the continuous supply of the Atlantic water mass at a steady rate. The EAG expanded meridionally and towards the WAG due to its interaction with a transient meander that developed off the Cape Tres Forcas. Therefore, both gyres tended to cover the entire meridional extent of the basin during their mature states. The Spanish boundary current proceeded mainly southward, crossing the basin near the eastern flank of the EAG and joined the Algerian coastal current. This southward current, together with the eastern flank of the EAG, formed the adjacent Almeria (Spain)–Oran (Algeria) front representing the eastward limit of the Alboran Sea circulation system [6].

The latter phase of the flow evolution also included some modifications within the interior of the WAG. As the Atlantic jet deflected more predominantly into the northeast, some Atlantic water from the strait entered directly into the WAG, in agreement with earlier studies [8], [37], [38]. A small relatively weak cyclonic eddy was formed within the center of the gyre, possibly due to the instability of the jet. Another stronger cyclonic eddy, located on the southern side of the gyre, may have originated from the closing branch of the WAG well before it reached the south coast by the strong curvature and westward advection of the jet. This type of gyre structure offered a variant of the Atlantic jet-WAG system [10].

**Figure 3:**
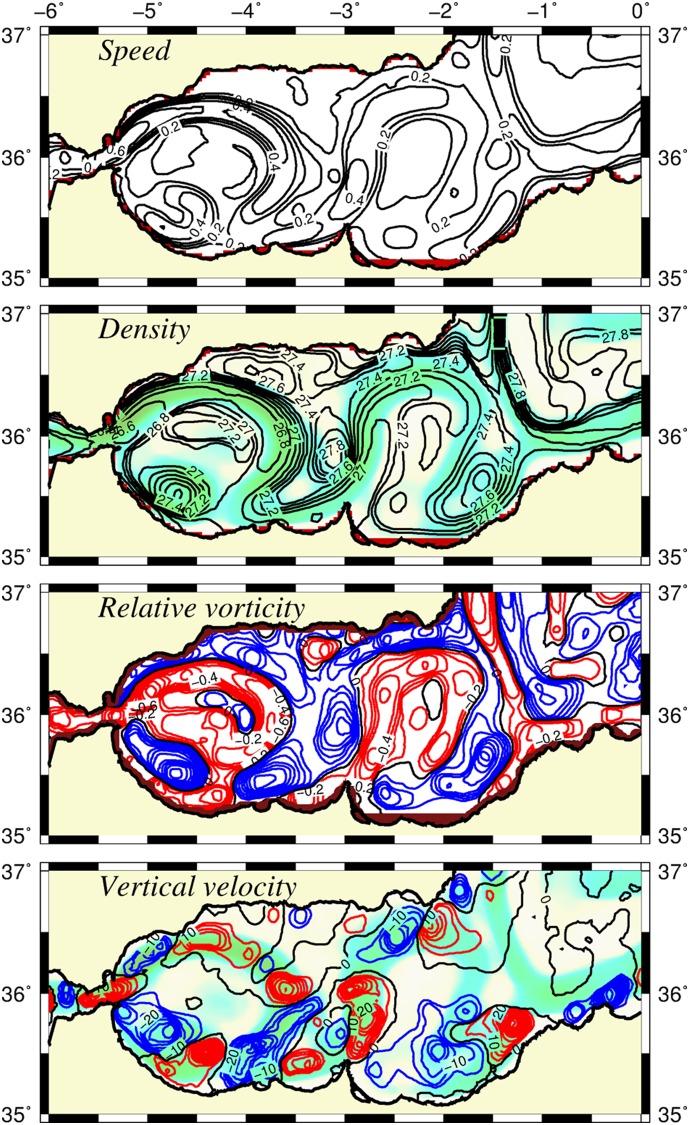
Main characteristics of the simulated flow field. Horizontal distributions of (a) the surface current speed (m s^−1^), (b) the surface density (kg m^−3^), (c) the vertical component of surface, relative vorticity (normalized by the planetary vorticity), and (d) the vertical velocity (m day^−1^) at 50 m depth. The background color shows the trajectory of the Atlantic jet. In the relative vorticity field negative (positive) vorticity values are shown in red (blue) color. Similarly, in the vertical velocity distribution, its upward (downward) component is shown in red (blue) color. The contour intervals are 0.1 for the vorticity, 5 m d^−1^ for the vertical velocity.

The surface density distributions accompanying the circulation fields in Fig. 2a–d show how the initial horizontally uniform dense water mass (∼27.8 kg m^−3^) was freshened and spatially structured up to the density range 27.0–27.5 kg m^−3^ by means of the steady inflow of Atlantic water. At day 120, the Atlantic jet attained a surface current speed of 0.7 m s^−1^ and a density of 26.5 kg m^−3^ near the Gibraltar exit section (Fig. 3a, b). They were modified to the ranges of 0.5–0.6 m s^−1^ and 26.5–26.8 kg m^−3^ around the WAG, whereas the surface density of cyclonic eddies inside the WAG exceeded 27.0 kg m^−3^. The Atlantic jet was separated from the ambient water mass by a well-marked narrow front identified by the density variations between 26.8 and 27.3 kg m^−3^ around the WAG. However, it appeared to lose much of its initial momentum and weakened by half around the EAG and further eastward along the coast (Fig. 3a). As the jet weakened, its density increased to the range 27.0–27.4 kg m^−3^ along the western side of the EAG and 27.2–27.4 kg m^−3^ along its eastern side, with respect to an interior density of ∼27.1 kg m^−3^. The southward Spanish current flowing along the eastern side of the EAG attained a speed of approximately 0.2 m s^−1^ and separated the Alboran surface water mass from the rest of the basin further east (Fig. 3b). The weakening of the Atlantic jet eastward was consistent with the gradual reduction of the zonal density gradient towards the eastern side of the model domain.

**Figure 4:**
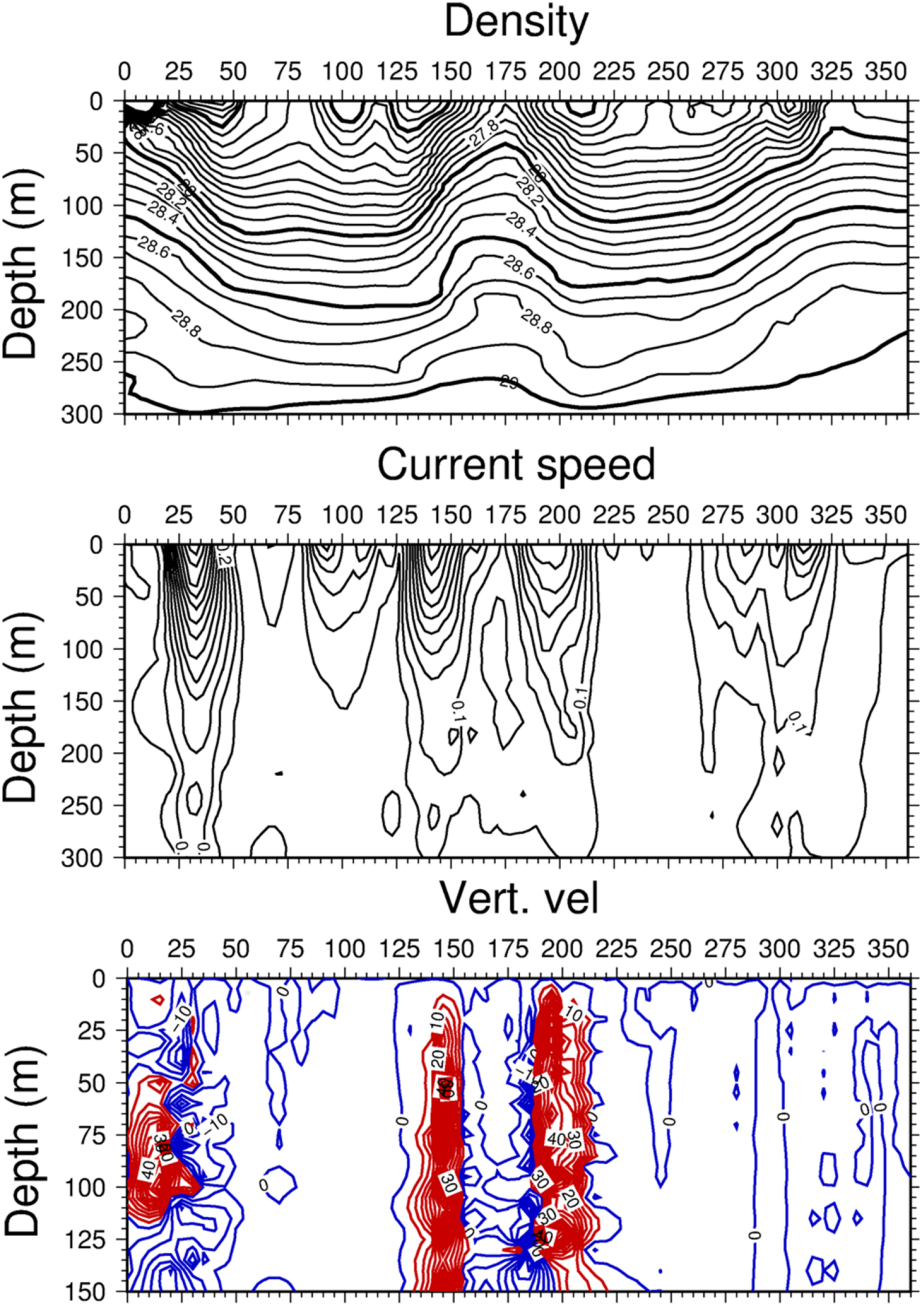
Cross section of the flow field. Vertical cross-sections of (a) density (kg m^−3^), (b) current speed (m s^−1^), and (c) vertical velocity (m day^−1^) along the transect shown in Fig. 2d. For vertical velocities, contours in red (blue) color represent upward (downward) velocities.

The vertical density structure of the circulation field is depicted by Fig. 4a for the transect that crosses the WAG diagonally and then extends zonally across the EAG towards the Algerian basin (see Fig. 2d). It identified the low density anticyclonic gyres with isopycnals in the range of ∼27.0–28.0 kg m^−3^ deepening towards their centers within the upper 100 m layer and to 28.5 kg m^−3^ between 150–200 m. The latter represented the interface between the Atlantic and Mediterranean water masses, in agreement with previous observations [8], [14]. Two frontal zones for both the WAG and the EAG were identified by the upward sloping isopycnals greater than 27.5 kg m^−3^ towards the surface. The vertical structure of the jet accompanying these well-marked narrow frontal zones extended to 200 m with a gradual reduction in its strength from 0.7 m s^−1^ at the surface around the WAG and 0.3–04 m s^−1^ elsewhere (Fig. 4b). As stated previously, the strength of the jet and its vertical dimension reduced eastward with the Almeria-Oran front being the weakest one.

### 2. Relative vorticity and vertical velocity fields

The surface distribution of the vertical component of the relative vorticity normalized by the planetary vorticity at day 120 (Fig. 3c) very closely resembled the surface flow and density fields (Fig. 3a, b). We recalled that the absolute value of this ratio is an estimation of the Rossby number; thus, it characterizes the degree of nonlinearity of the flow field. The circular path of the Atlantic jet around the WAG was related to the generation of an anticyclonic relative vorticity (Fig. 3c) due to the upward tilting of the interface and compression of the upper layer flow along the strait [10], [32]. The jet around the gyres was surrounded by several cyclonic eddies with positive relative vorticity and dense water patches. The eddy closest to the exit section was associated with the strong density gradient that set a strong vertical geostrophic shear and thus the generation of cyclonic vorticity [10]. This cyclonic vorticity deflected the Atlantic jet towards the northwestern coast. Further downstream, the outer flank of the jet generally featured a strong relative vorticity gradient from negative (around −0.5 on the inner side) to positive (up to +0.5 on the outer side) in agreement with previous observations [39]. The Rossby number reached values over 0.5 along the jet, which indicated its nonlinear character and the relevance of ageostrophic motion.

The vertical velocity distribution at 50 m indicated patches of positive (upward) and negative (downward) vertical motions with velocities as high as 50 m day^−1^ (Fig. 3d). They were linked to the strong vorticity centers because the conservation of potential vorticity states that the increase in the relative vorticity from the meander crest to the subsequent trough of the frontal zone needs to be accompanied by a downwelling motion. Conversely, the frontal zone from the meander trough to the crest, having a decrease of the relative vorticity, involved an upwelling motion. Although the marked nonlinearity of the flow structure hindered the identification of the meander crests and troughs precisely, Fig. 3c, d shows a close association of the strong cyclonic vorticity centers with the strong downward velocities and the anticyclonic centers with upward velocities. This type of flow structure provided a clear indication of the frontogenesis taking place as a result of breaking down the geostrophic balance of the along-front current in the presence of strong cross-frontal density gradients. High velocity shears and the development of ageostrophic cross-frontal circulation cell restored the geostrophy. Providing further details on the dynamics of frontogenesis is beyond the scope of the present study, and we refer to the existing theoretical studies [40]–[49]. We noted that the regions characterized by the quasigeostrophic dynamics differed markedly from those of the ageostrophic dynamics in Figs. 3c, d. For example, the centers of the anticyclonic gyres acquired much weaker anticyclonic relative vorticity (<0.3) and weaker (<5 m day^−1^) downward (instead of upward as in the ageostrophic regions) vertical velocities.

### 3. Biogeochemical characteristics

**Figure 5:**
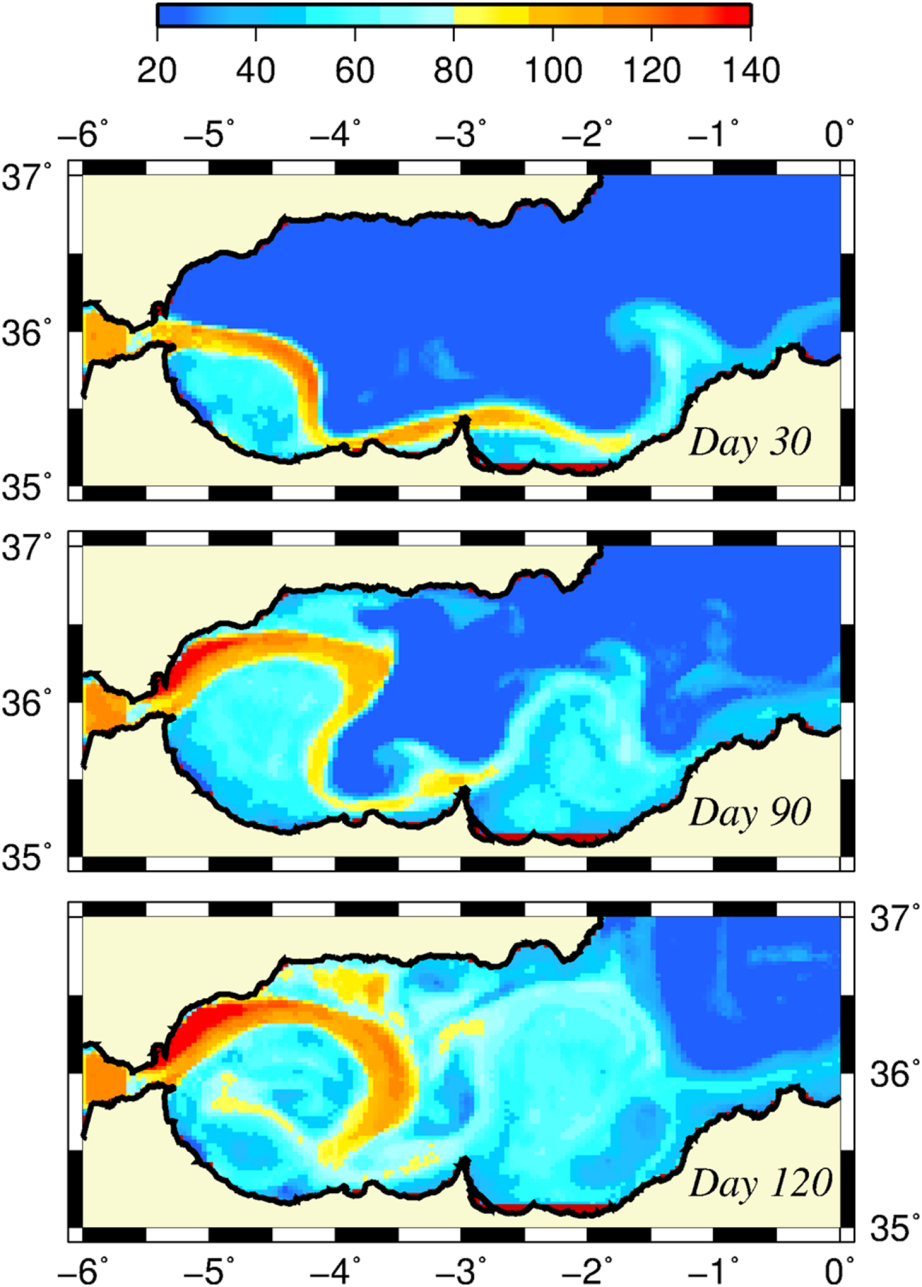
Modeled nutrient concentrations. Horizontal distribution of depth integrated nitrate distributions over 75 m layer (mmol N m^−2^) at days 30 (a), 90 (b), and 120 (c).

**Figure 6:**
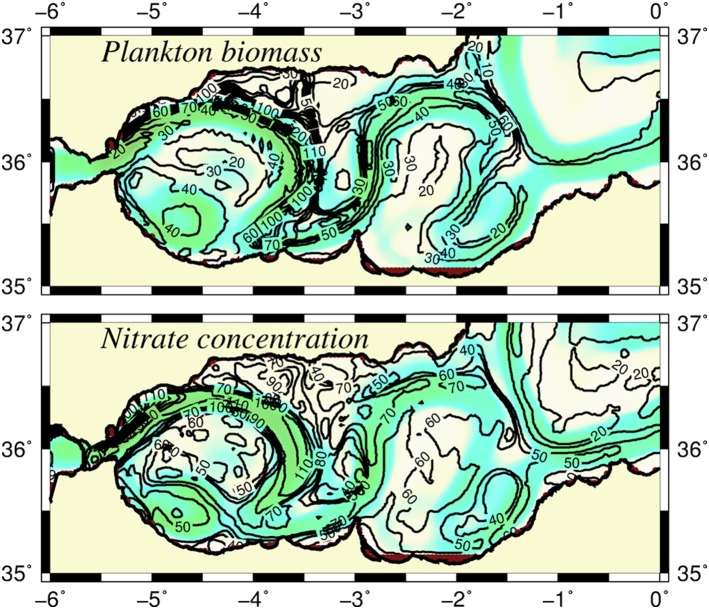
Modeled plankton biomass. Horizontal distributions of (a) integrated plankton biomass (phytoplankton plus zooplankton) and (b) integrated nitrate concentration over the upper 75 m layer (mmol N m^−2^), The background color shows the trajectory of the Atlantic jet. The contour interval is 10 mmol N m^−2^ for both plankton biomass and nitrate concentration.

Starting initially from nutrient-starved conditions and maintaining the absence of horizontal nutrient transport from the Gibraltar Strait, the euphotic layer-integrated nitrate distributions (shown in Fig. 5a–c) illustrated how the developing frontal jet-gyre circulation system built up nutrients within the upper 75 m layer (roughly representative of the winter euphotic zone). The region to the east of the Almeria-Oran front remained nutrient depleted. Nutrients first accumulated along the south coast (Fig. 5a) and then gradually spread over the basin by the meandering jet and, at the same time, by their recycling within the upper layer water column (Fig. 5c). The structural changes that took place in the nutrient distributions indicated the efficiency of the frontal dynamics for supporting enhanced biological production in the Alboran Sea, taking into account that the model excluded the lateral nutrient supply from the Gibraltar Strait. The corresponding depth integrated plankton (the sum of phytoplankton and zooplankton) biomass at day 120 indeed revealed relatively high values up to 100 mmol N m^−2^ inside the meandering jet around the WAG and roughly half of it around the EAG (Fig. 6a). They were well-correlated with the nutrient accumulation in excess of 100 mmol m^−2^ around the WAG and approximately 60–70 mmol m^−2^ around the EAG (Fig. 6b). Nutrient concentrations approximately 50 mmol m^−2^ within the central parts of the gyres should be related to their accumulation due to isopycnal subduction from the peripheries. Owing to the ageostrophic frontal dynamics, the coastal strip to the north of the Gibraltar junction region, as well as the northern coastal zone confined between the WAG and the Spanish coast, also acquired relatively high plankton biomass. On the contrary, the biomass within the central parts of the gyres and in the cyclonic region between the gyres and along the south coast remained below 30 mmol m^−2^ (Fig. 6a). The Algerian basin to the east of the Almeria-Oran front exhibited even more oligotrophic character. All of these regions involved relatively low integrated nitrate concentrations in response to the weaker vertical velocities on the order of only few m day^−1^ (Fig. 3d) associated with the quasigeostrophic motion.

**Figure 7:**
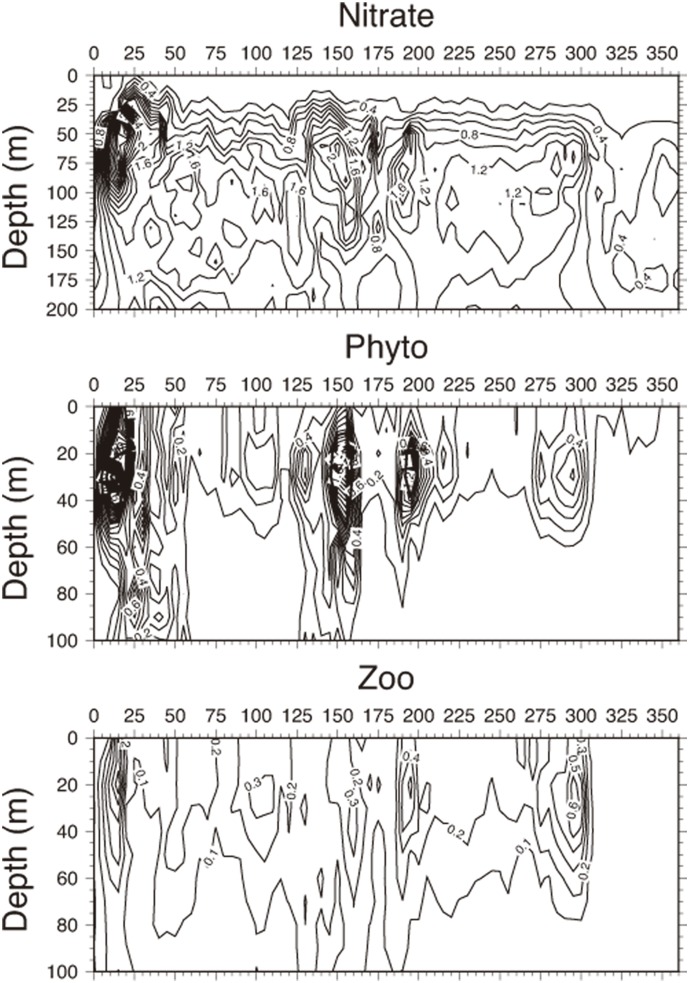
Cross section of biological tracers. Vertical cross-sections of (a) nitrate concentration, (b) phytoplankton biomass, and (c) zooplankton biomass expressed in mmol N m^−3^ along the transect shown in Fig. 2d.

The zonal nitrate cross-section for the transect shown in Fig. 2d indicated maximum concentrations of 3.0 mmol N m^−3^ and 2.0 mmol N m^−3^ at the northwestern and eastern flanks of the WAG, respectively (Fig. 7a). They occupied subsurface levels centered at 25 km and 140 km from the origin in Fig. 7a. The upward compression of the nitracline in these regions indicated a higher nitrate input towards the surface by the relatively high vertical velocities (Fig. 4c). This feature also developed with a slightly weaker efficiency around the EAG periphery (centered at 190 km and 290 km). Relatively high concentrations of approximately 1.5 mmol N m^−3^ within the central parts of the WAG and 1.2 mmol N m^−3^ within the EAG at depths between 75 and 175 m were likely related to the isopycnal subduction of upwelled nutrients, as well as those arising from the organic matter recycling. On the contrary, the narrow cyclonic zone confined between the eastern flank of the WAG and the western flank of the EAG reflected the strong subduction associated with the downward motion, leading to a pronounced reduction in nitrate concentrations (Fig. 4c). This region was also deficient in phytoplankton biomass, whereas its higher concentrations coincided with those of nitrate in the frontal regions (Fig. 7b). The phytoplankton biomass was much higher and extended deeper around the WAG than around the EAG, which was consistent with the nitrate transect.

**Figure 8:**
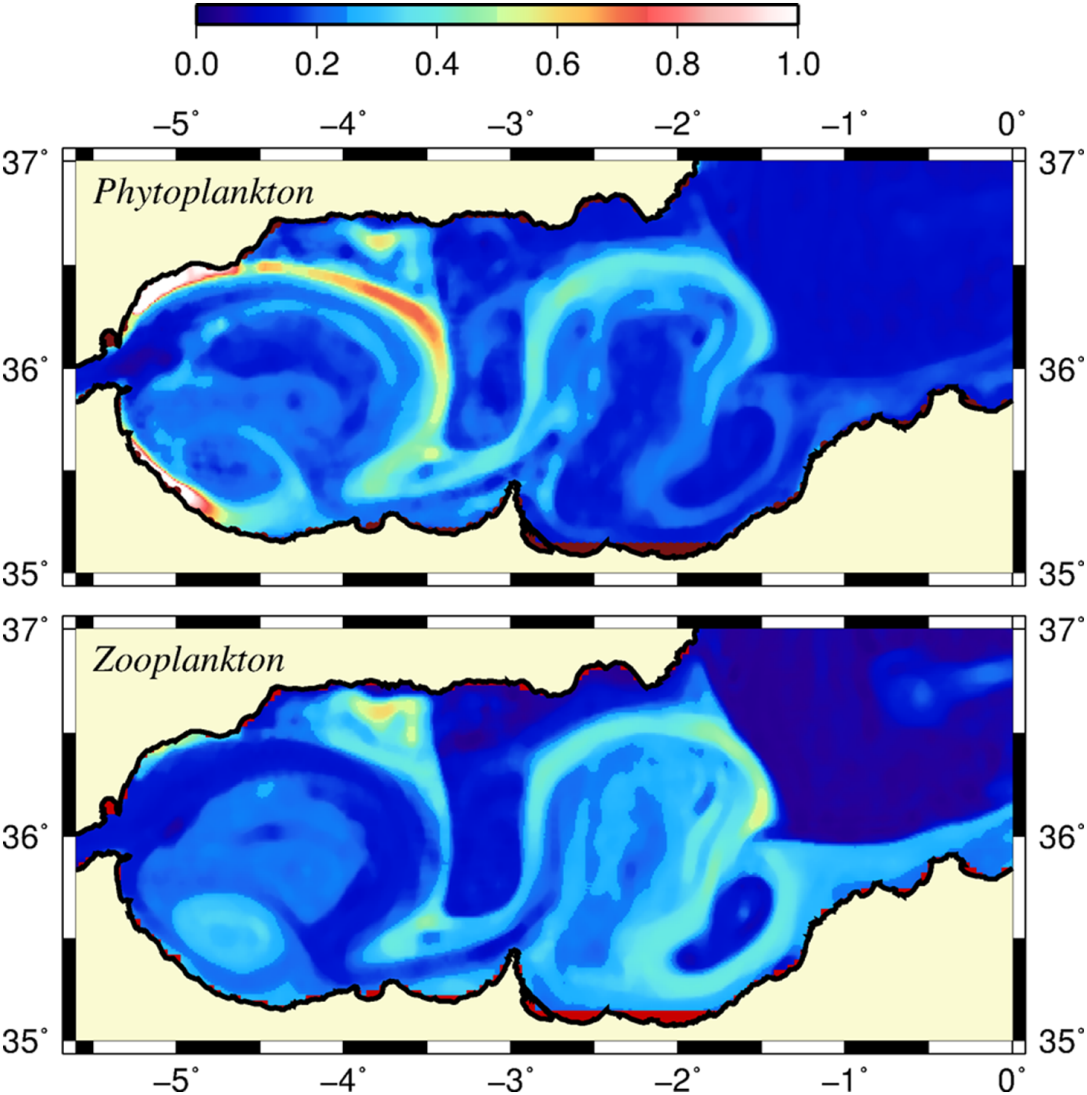
Surface phytoplankton and zooplankton biomass. Horizontal distributions of (a) surface phytoplankton biomass and (b) surface zooplankton biomass (mmol N m^−3^) at day 120.

An interesting feature of the narrow frontal region of approximately 15–20 km width along the jet trajectory revealed opposite phytoplankton and zooplankton biomass distributions. For example, the surface phytoplankton biomass was highest at the frontal zone of the WAG (Fig. 8a) but the corresponding zooplankton biomass was much lower (Fig. 8b). The inefficiency of the secondary production (with respect to the primary production) in this region was apparently related to the adverse effect of strong along-front advection of the phytoplankton biomass. The reverse situation occurred at the Almeria-Oran frontal zone where the horizontal current was relatively weak and the ageostrophic dynamics was less effective. Thus, zooplankton may exert a more efficient grazing pressure on phytoplankton and present a biomass comparable with that of phytoplankton.

## Discussion and Concluding Remarks

The performed simulation from the biophysical model linked the efficiency and persistency of the autotrophic frontal production to the meandering Atlantic jet of the upper layer circulation system of the Alboran Sea. The simulation started initially with nutrient-starved conditions, it excluded the horizontal nutrient transport from the Gibraltar Strait and also the likely modifications of ageostrophic vertical motion from other physical processes, such as wind-driven vertical advection and turbulence, and enhanced buoyancy-induced mixing by strong winter cooling. Identifying the relative contributions of all of these processes remains to be explored in future studies.

The jet-front system was set up and maintained by the flow and stratification characteristics prescribed at the western inflow section. The Atlantic jet entered from the Strait of Gibraltar at a fixed rate in the form of a buoyant surface intensified current in the absence of sub-inertial (weekly to monthly) variability in response to changing atmospheric pressure and wind patterns of the region. The jet remained stable and coherent without mesoscale-submesoscale variability in response to the frontal instabilities [5], [7], [12], [22], [37], [38], [54], [57], [58].

Up on issuing from the Gibraltar Strait, the Atlantic jet deflected northeastward, looped around the WAG and proceeded eastward in the form of a boundary current along the North African shelf break/slope topography. Its meandering around the WAG was imposed mainly by the generation of anticyclonic relative vorticity due to the upward tilting of the interface and compression of the upper layer flow along the strait. Another intense meander developed along the African coastline in response to perturbations developed in the boundary current triggered by the coastline irregularities and the topographic beta effect. In our model, the continuous supply of a fixed rate of Atlantic water mass from the Gibraltar Strait caused the WAG to cover the entire western Alboran basin in its mature state. In reality, its growth is modulated by the subinertial variability of the upper layer transport across the strait and by the impacts of the regional atmospheric conditions on the Alboran Sea circulation system. Despite such variability, the northeastern expansion of the WAG and the presence of its front near the northern coast is a persistent feature of the Atlantic jet-WAG system as it is corroborated, for example, by the seasonal surveys carried out in 1996–97 in this region [14], [50]. The mature state of the flow structures developed in our simulation resembles the one observed during December 1996 [9].

The meandering current, involving strong shear vorticity (scaled by the planetary vorticity) with values greater than 0.5 in some places, was accompanied by a well-defined and narrow frontal zone with its structure evolving on daily-to-weekly time scales. The nonlinearity of the jet broke down the geostrophic balance of the along-front current and developed secondary cross-front ageostrophic motions that supported intense localized upwelling from the meander troughs to the crests on the right (anticyclonic) side of the front looking downstream. The compensatory downward motion occurred from the crests to the troughs on the cyclonic (left) side of the front. The magnitude of vertical velocities up to 50 m day^−1^ agreed with the estimated velocities deduced from data collected in different hydrographic field surveys using the Quasi-Geostrophic approximation [7]–[9], [51], although the small-scale patterns are much complex in the present study.

The upwelling centers, associated with the ageostrophic motion, supplied nutrients into the euphotic layer at a rather high rate and supported relatively high primary productivity. The resulting plankton biomass was higher at subsurface levels due to stronger vertical motion at greater depths, an observation that was supported by the winter observations at the Almeria-Oran front [17]. This feature implied a more efficient biological production capacity of the Alboran Sea than suggested by its surface signature. A similar result was proposed recently by Macias et al. [21] for the whole Mediterranean as well. The compensatory downward motion from the crests to the troughs limited the production by subducting nutrients toward deeper levels. The strong along-front jet distributed phytoplankton and nutrients away from the localized production centers to the rest of the frontal circulation system while they were subsequently consumed by zooplankton. On the contrary, the centers of the anticyclonic gyres and the offshore surface Mediterranean waters remained oligotrophic with severe nutrient depletion. The ageostrophic dynamics promoted more intense plankton production on the western side of an anticyclonic meander (e.g., the EAG) compared to its eastern side (e.g., the Almeria-Oran front) because of the preferentially strong upward motion of the former region (from trough to crest). The weakening of the ageostrophic processes eastward [8] reduced the efficiency of the primary production in agreement with the observations of relatively low chlorophyll concentrations (∼1.0 mg l^−1^) within the Almeria-Oran frontal zone of the EAG [17] with respect to much higher concentrations (>3.0 mg l^−1^) within the Atlantic jet-front system at the northern flank of the WAG [13]. In the case that the WAG was sufficiently large, the ageostrophic motion contributed to relatively high plankton production along the coastal strip to the north of the Gibraltar junction region, as well as the northern coastal zone confined between the WAG and the Spanish coast, in addition to the wind and eddy-induced upwelling processes [14], [50], [52]–[54]. These features were indeed supported by the surface chlorophyll distribution shown in Fig. 1, as well as additional examples given by Garcia-Gorriz and Stips [24].

The available *in situ* observations may support the fact that the model replicated the dominant mechanism underlying plankton productivity in the region. For example, observations performed along a meridional transect extending from the Spanish coast to the center of the WAG during July and December 1996 [14], [50] identified the frontal zone of the Atlantic jet bordering the northern flank of the WAG with relatively high geostrophic currents up to 0.75 m s^−1^ and chlorophyll concentrations up to 3 mg l^−1^ compared to concentrations less than 0.5 mg l^−1^ within the gyre interior. A similar survey performed during May 1998 [13] reported even higher chlorophyll concentrations up to 6 mg l^−1^ within the frontal jet zone. The May 1991 observations [26] along a north-south transect crossing the AEG meridionally also identified the frontal zone at the northern periphery of the EAG by the upsloping of the nitracline, higher concentrations of chlorophyll-a, and particular organic nitrogen with respect to adjacent waters. As in the present study, these features were explained qualitatively in terms of the cross-frontal secondary circulation [15], [26]. Moreover, the primary production along the Almeria-Oran front on the eastern flank of the EAG was found to be 2.5 times higher when compared to the more oligotrophic surrounding waters during the same observations [55]. They were also supported by 10 to 100 times higher vertical particle fluxes measured at 100 m and 300 m depths [56]. In situ measurements [16], [17] have also detected anomalously high chlorophyll-a concentrations associated with the Almeria-Oran front along the eastern flank of the EAG.

## Extended Model Description

### 1. Biological model

The NPZD model equations for the nutrient (N), phytoplankton (P), zooplankton (Z), and detritus (D) compartments are expressed in the general form by.
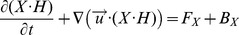
(A1)where X denotes each of the state variables, **u** is the three dimensional fluid velocity, H = h + η the total water depth with h defining the bottom topography and η the surface elevation, F_X_ denotes the sum of horizontal and vertical diffusion terms represented similar to those in the physical model [25], and B_X_ denotes the biological source-sink terms that are expressed for each model compartment by [43]

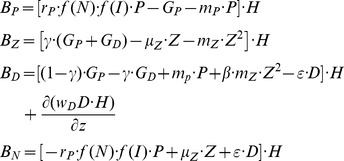
(A2a<?ENTCHAR hyphen?>d)where r_P_ = 1. 3 day^−1^ denotes the maximum phytoplankton growth rate, m_P_ = 0.06 day^−1^ and m_Z_ = 0.10 day^−1^ the phytoplankton and zooplankton mortality rates, respectively, µ_Z_ = 0.05 day^−1^ the zooplankton excretion rate, γ = 0.7 the assimilation efficiency of grazing, and ε = 0.25 day^−1^ the remineralization rate. In A2b,c, the zooplankton mortality is parameterized in the quadratic form for the model closure and the stability reasons. It incorporates both the natural mortality loss and the loss due to predation. The parameter β in A2c representing the recycling efficiency of zooplankton loss is set to 0.5, suggesting 50% of the zooplankton mortality lost permanently by the higher level predations, whereas the other 50% is due to natural mortality recycled within the system. Moreover, f(N) and f(I) denote the nutrient and light limitation terms of primary productivity, respectively, and are parameterized by.
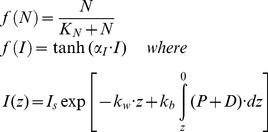
(A3a<?ENTCHAR hyphen?>c)where K_N_ = 0.5 mmol N m^−3^ denotes the half saturation constant for the nitrate uptake of phytoplankton; α_I_ = 0.03 (W m^−2^)^−1^ is the initial slope of the light limitation curve; k_w_ = 0.05 m^−1^ is the background value of the light extinction coefficient; k_b_ = 0.04 mmol N^−1^ m^2^ is the additional contribution due to water turbidity; and I_s_ = 40 W m^−2^ is the surface winter-mean photosynthetically available radiation. Considering the fact that the euphotic layer during winter was characterized steadily by low temperatures of approximately 15°C, the temperature limitation was not represented explicitly but was indirectly introduced by taking a lower phytoplankton growth rate.

G_P_ and G_D_ are the grazing terms of zooplankton on phytoplankton and detritus, respectively, as expressed by.
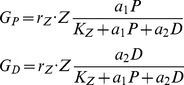
(A4a,)where r_Z_ = 0. 5 day^−1^ the maximum zooplankton grazing rate, K_Z_ = 0.5 mmol m^−3^ the half saturation constant of zooplankton grazing, a_1_ and a_2_ refer to the food preference coefficients of zooplankton on the phytoplankton and detritus consumptions, respectively. In the present simulations we set a_1_ = 1.0 (100% efficiency on phytoplankton feeding) and a_2_ = 0.5 (50% efficiency on detritus feeding). The sinking velocity of detritus was expressed by.
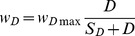
(A5)


where S_D_ = 0.2 mmol m^−3^ half saturation constant, and w_Dmax_ = 8.0 m day^−1^ is the maximum sinking velocity. A5 suggests faster sinking of detritus at higher concentrations. The parameters were optimized by a series of sensitivity experiments based on their values used by the one-dimensional model simulations for the same region [29]. According to this parameter setting, the phytoplankton group was better adapted to high nutrient, low light conditions within a weakly stratified water column and generally represented diatoms.

### 2. Model implementation

The original POM model was modified by the implementation of the fourth-order pressure gradient algorithm to reduce the internal pressure gradient error [59]–[61]. The bottom topography, retrieved from the one minute bathymetry data set [62] (available at http://topex.ucsd.edu/cgi-bin/get_data.cgi), was mapped into the model domain using bilinear interpolation. The topography has a complex structure with sharp gradients near the coastal boundaries but its main feature is its shoaling from about a 2000 m depth on the eastern side of the model domain to 500 m near the Gibraltar entrance (Fig. 1). The sharp topographic gradients defined by the slope parameter *r = *Δ*h/h* were smoothed to make *r* less than 0.5; *h* representing the mean depth of two adjacent grid points and Δ*h* their difference. The vertical viscosity and diffusivity were estimated by the 2.5 level Mellor-Yamada turbulence energy model [63]. Their minimum values were set to 2×10^−5^ and 1×10^−6^ m^2 ^s^−1^, respectively. The horizontal friction terms in the momentum equations were parameterized in the Laplacian form and the horizontal viscosity was defined by the sum of a constant value of 20 m^2 ^s^−1^ and a flow dependent contribution provided by the Smagorinsky parameterization. The horizontal diffusivity was set to half of the horizontal viscosity. The external (depth-integrated) and internal (baroclinic) modes of the equations were solved using time steps of 3 seconds and 150 seconds, respectively.

### 3. Initial and boundary conditions

The circulation model was initialized at the state of rest and by the horizontally uniform water mass that represented the Medar/Medatlas climatology winter mean conditions. The temperature range was 15.8–14.4°C and the salinity range was 37.6–37.75 psu within the upper 100 m layer. The temperature gradually changed to 13.0°C and the salinity changed to 38.5 psu at 250 m depth, where it met the slightly warmer and more saline water mass of the Levantine Intermediate Layer (∼13.2°C and ∼38.55 psu) at depths between 300–600 m with respect to the deep water mass (12.8–12.9°C and ∼38.45 psu) underneath. At the western open boundary, the relatively warmer (17°C) and less saline (35.5 psu) Atlantic water mass was prescribed within the upper 200 m layer, whereas the deeper levels comprised the Mediterranean water mass with 13°C and 38.5 psu. Moreover, an inflow with 37.3 psu salinity within the upper 200 m layer of the water column, where bottom depth was shallower than 500 m, was prescribed across the northern boundary (corresponding to the Ibiza channel at 39°N latitude) to incorporate the likely effects of the southward flowing Catalano-Balearic coastal current on the Alboran Sea circulation.

The wind stress and the total heat flux were set to zero. The latter was specified by setting a 80 Wm^−2^ cooling rate due to the sum of long wave radiation, the latent and sensible heat flux components, and 80 W m^−2^ warming due to the short wave radiation. The neglect of the atmospheric forcing excluded the likely effects of the wind and buoyancy-induced vertical mixing, as well as the coastal upwelling/downwelling events on the plankton production. Photosynthetically available radiation was taken from half of the short wave radiation (i.e., 40 W m^−2^) throughout the integration period. At the bottom, adiabatic boundary conditions were applied to the diffusive fluxes of temperature, salinity, and biological fields. The momentum flux was formulated in terms of the quadratic bottom friction by combining the velocity profile with the logarithmic law of the wall.

For the western and northern open boundaries, the values of temperature and salinity were evaluated from the adjacent interior grid points during the outflow conditions and were set to those of the prescribed boundary conditions during the inflow conditions. Along the eastern open boundary, the latter was replaced by setting the boundary values by their values provided by the previous time step that decouples the temperature and salinity fields within the basin from rest of the Western Mediterranean thermohaline structure.

The specification of the inflow/outflow conditions across the boundaries followed the standard setting given by the POM. The depth averaged currents of 3.0 cm s^−1^ and 1.0 cm s^−1^ (the barotropic transports) were specified in the form of Orlanski type forced radiation condition along the western open boundary and the coastal part of the northern open boundary at depths shallower than 500 m. The inflow conditions were kept unchanged for the integration period of the model, indicating that it neglected the temporal modulation of the transports due to the tidal and sub-inertial oscillations across the Gibraltar Strait. Their free radiation condition versions were imposed for the rest of the northern boundary and the eastern boundary. The total flow (the baroclinic flow) across the open boundaries was treated by the free radiation conditions and were computed by the evolving baroclinic structure of the flow field at each time step in response to the forcing with the barotropic transports. This setting implied that the model did not specify a priori the upper and lower layer transports (but their difference was equal to the imposed barotropic transport) at the western open boundary.

For the biological fields, the horizontal advection was switched off at three grid points nearest to the northern and eastern open boundaries to avoid their contamination by numerical noise. Their values along these boundaries were set to their nearest adjacent values. Along the western open boundary, the biological fields were set to their typical observed values. This setting was not critical because we later switched off the advection process of the biological fields along the Gibraltar Strait to decouple the role of the frontal jet on the production from an additional contribution by the lateral supply of nutrients and biogenic material from the Gibraltar Strait. In addition, the initial mean nitrate profile was modified by subtracting 1.5 mmol m^−3^ at all depths to reduce the nitrate content of the upper 100 m layer to zero. Therefore, all of the available nitrate supporting the plankton production within the upper layer water column originated solely from the diapycnic advective fluxes from deeper levels without any a priori contribution of the initial nitrate availability.
