# Correction: Fueling Plankton Production by a Meandering Frontal Jet: A Case Study for the Alboran Sea (Western Mediterranean)

**DOI:** 10.1371/journal.pone.0116653

**Published:** 2014-12-26

**Authors:** The PLOS ONE Staff

There is an error in affiliation 3 for Diego Macias. Affiliation 3 should be: European Commission, Joint Research Centre, Institute for Environment and Sustainability, Ispra, Italy.
